# Supplementary figures and images for: EMT network-based feature selection improves prognosis prediction in lung adenocarcinoma
Source: PLoS One. 2019 Jan 31;14(1):e0204186. doi: 10.1371/journal.pone.0204186 (PMC6354965; doi:10.1371/journal.pone.0204186)

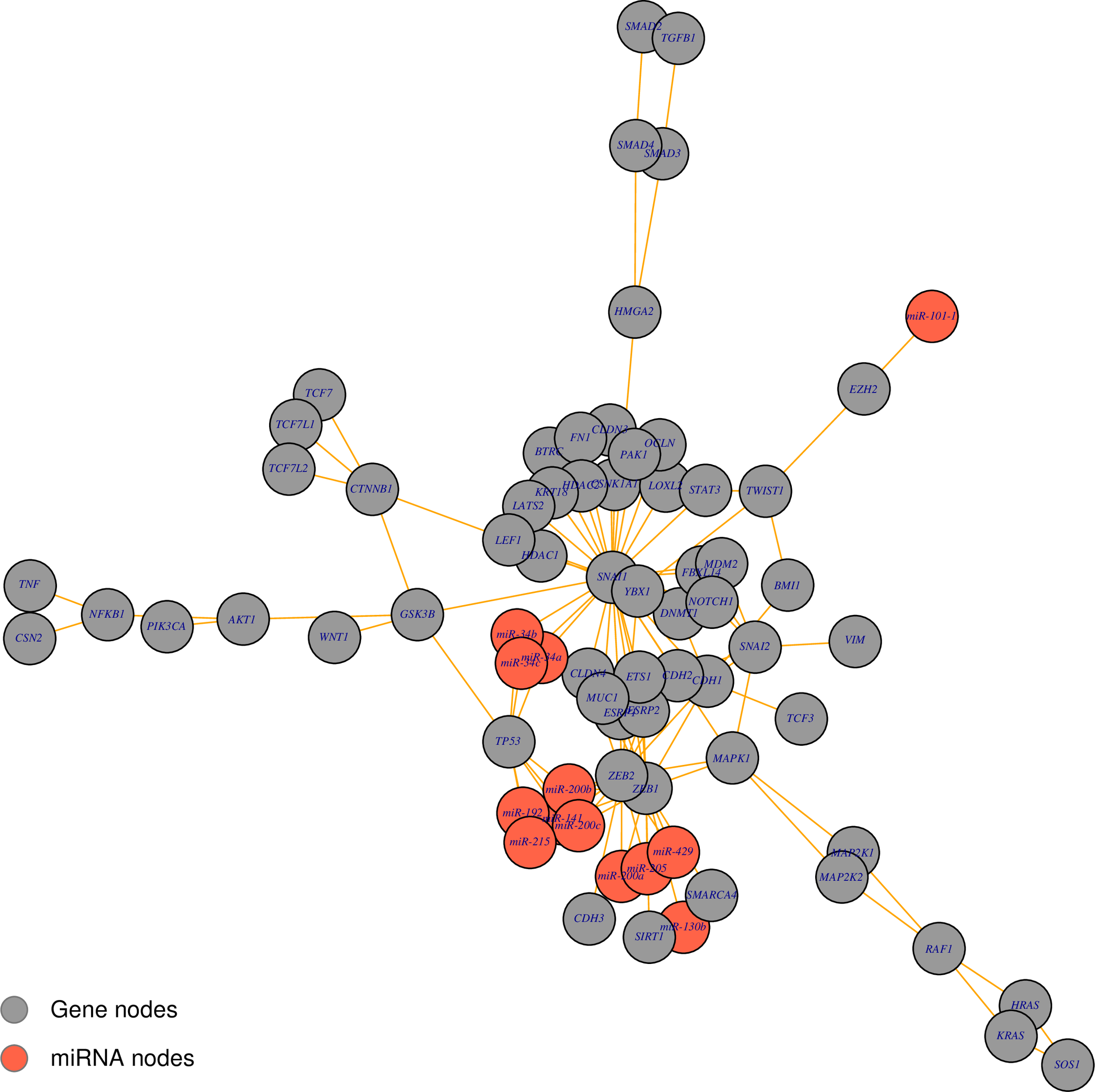

Supplement: S1 Fig — The names of genes and miRNAs are given on the nodes. (TIF) [file pone.0204186.s001.tif]

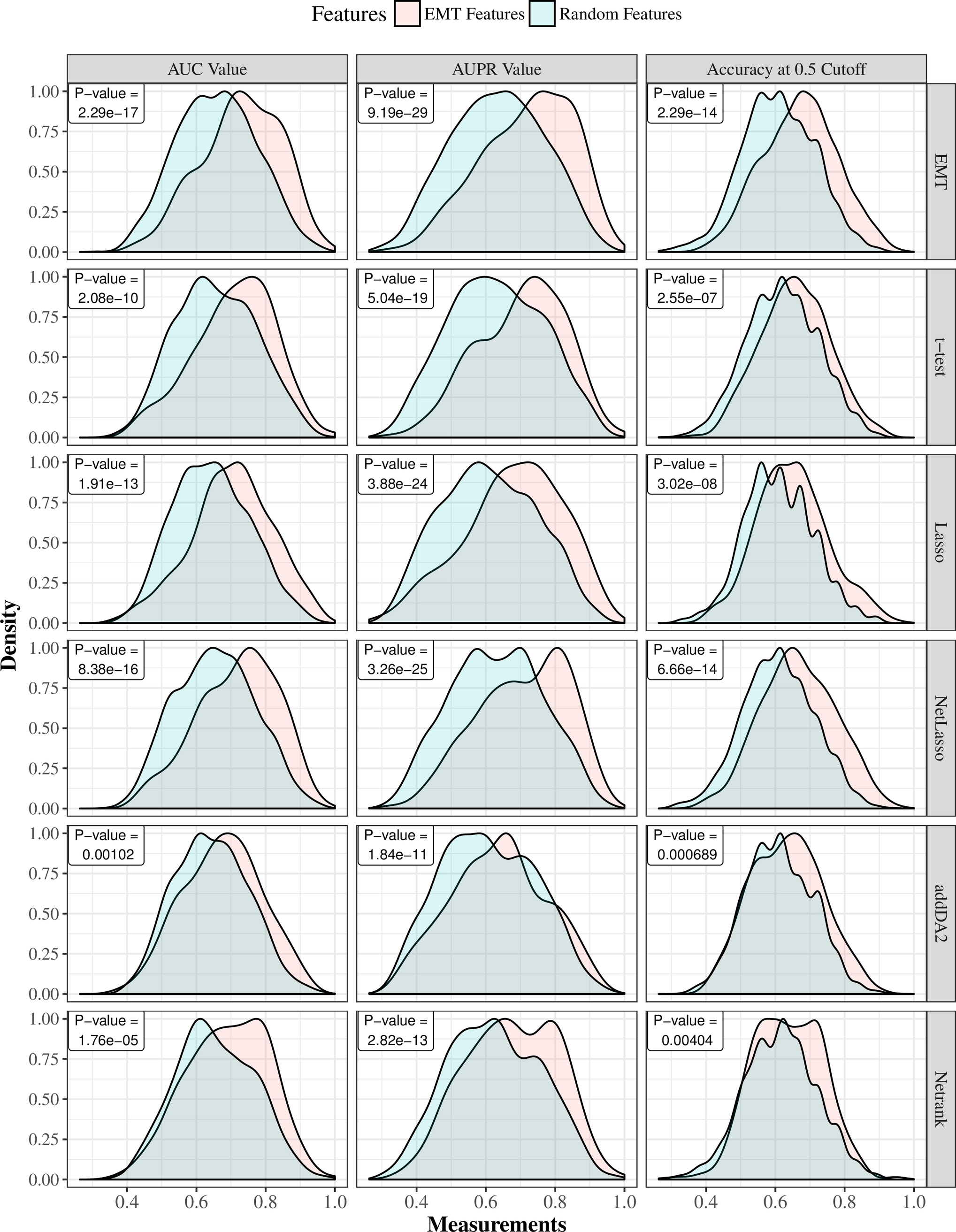

Supplement: S2 Fig — Gaussian kernel is used to estimate the density functions based on results from 30 times 10-fold cross-validation. For each cross-validation fold, EMT features and random features are tested on the same training and testing samples. The comparisons on five feature selection algorithms together with the comparative group of using all EMT features are shown. The p-values of paired t-tests are provided. (TIF) [file pone.0204186.s002.tif]

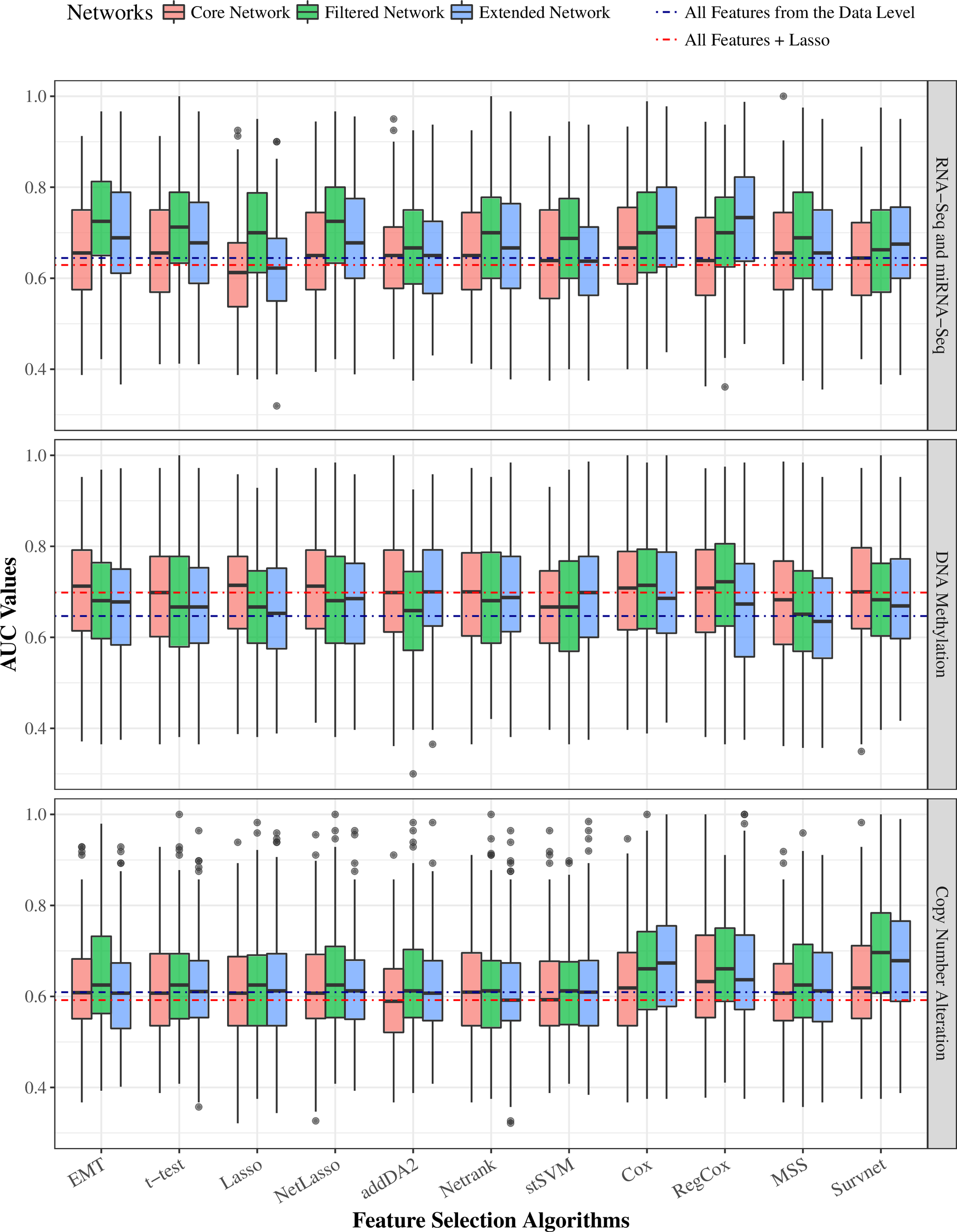

Supplement: S3 Fig — The three panels correspond to three data levels. Within each panel, the AUC values of the 10 algorithms are plotted. Each algorithm has three boxes of different colors denoting the 3 EMT networks. The blue and red dotted lines within each panel are the median AUC values of two comparative groups: 1) using all data level features and 2) Lasso feature selection on all data level features. (TIF) [file pone.0204186.s003.tif]

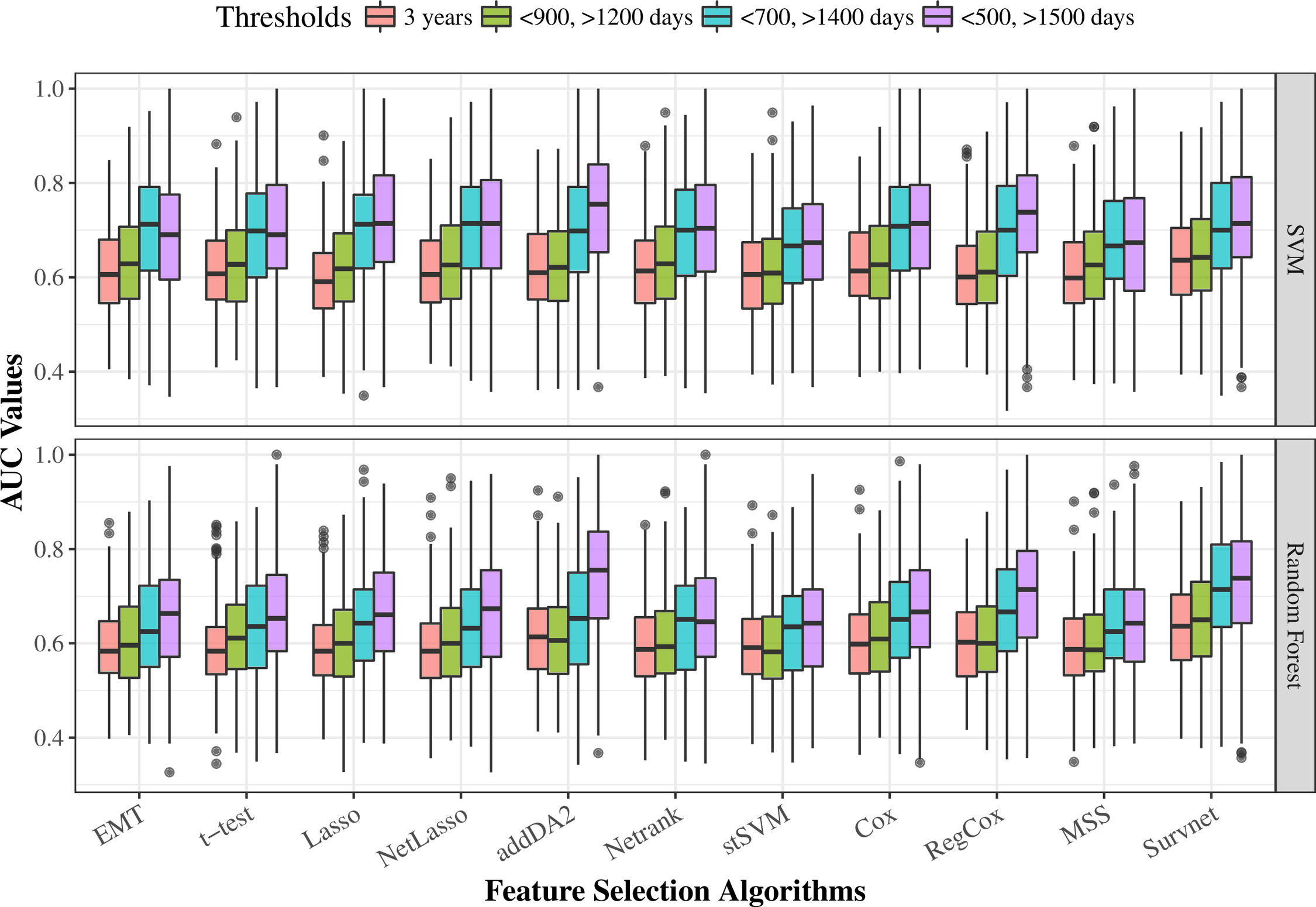

Supplement: S4 Fig — The data level is DNA methylation data. The network is EMT core network. (TIF) [file pone.0204186.s004.tif]

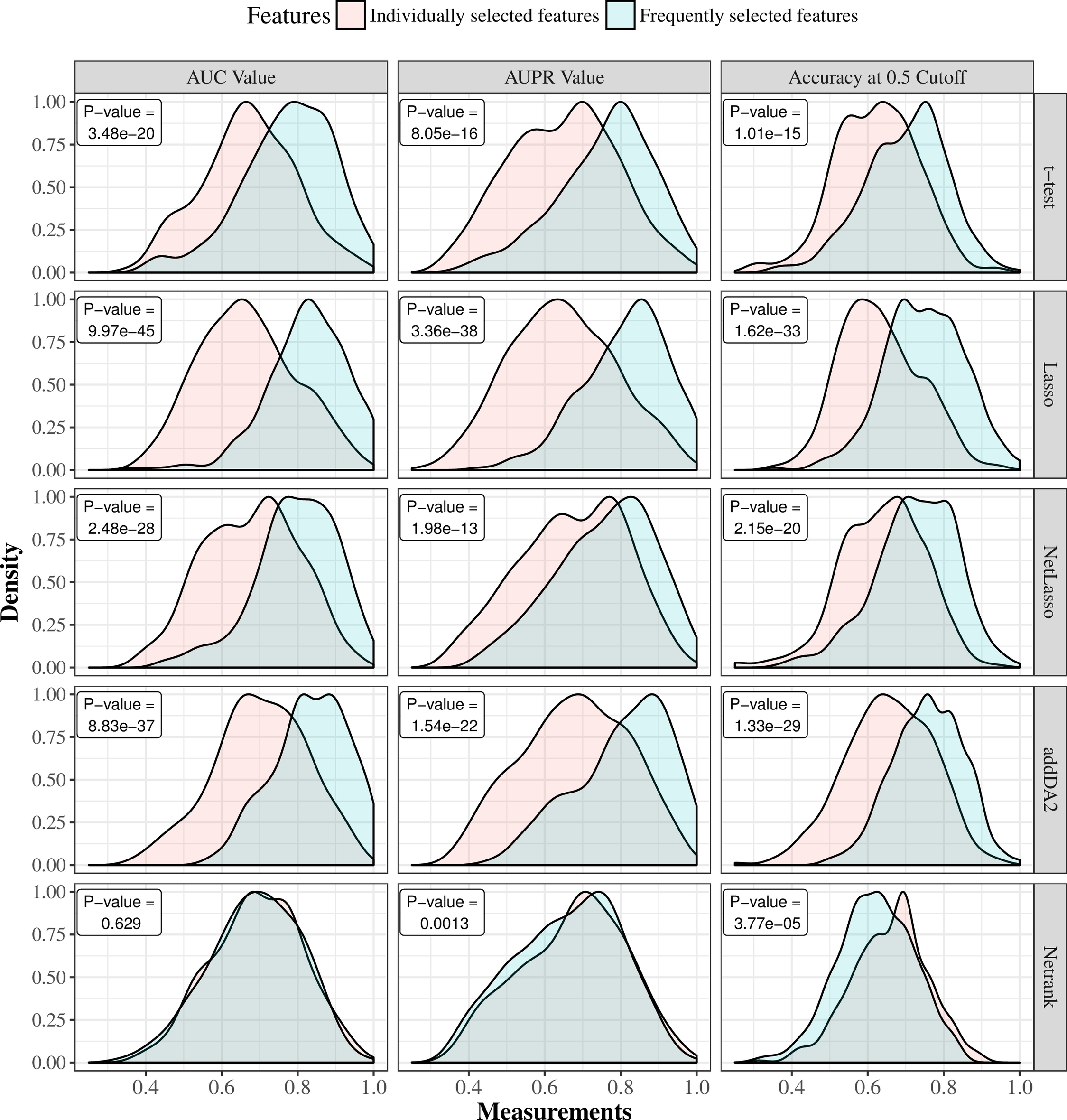

Supplement: S5 Fig — We used DNA methylation data and extended EMT network for feature selection and SVM classifier for classification. Gaussian kernel is used to estimate the density functions based on results from 30 times stratified 10-fold cross-validation. For each cross-validation iteration, individually selected features and FSFs are tested on the same training and testing samples. The comparison between the two feature groups is shown on five feature selection algorithms together with the p-values of paired t-tests. (TIF) [file pone.0204186.s005.tif]

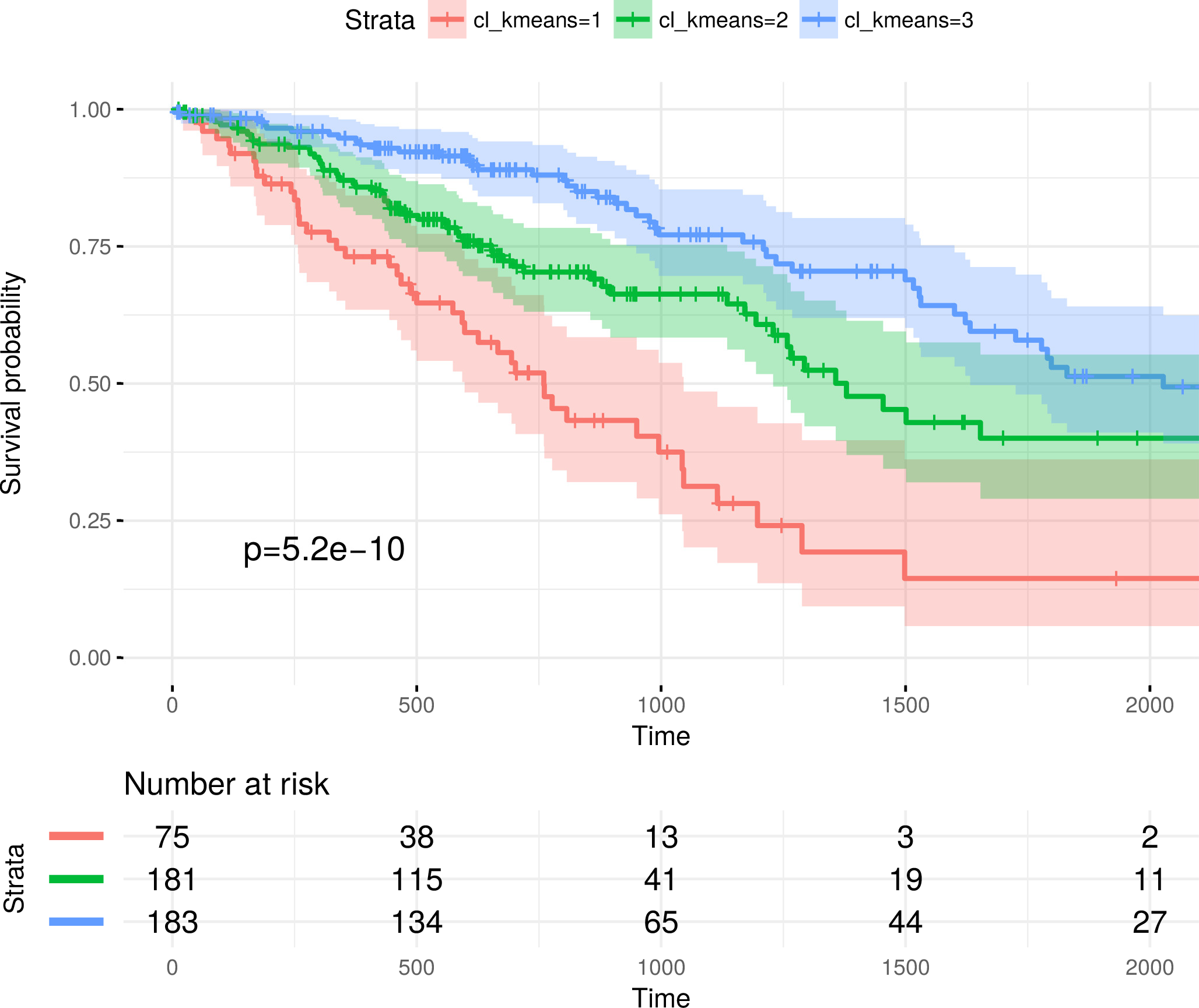

Supplement: S6 Fig — (TIF) [file pone.0204186.s006.tif]

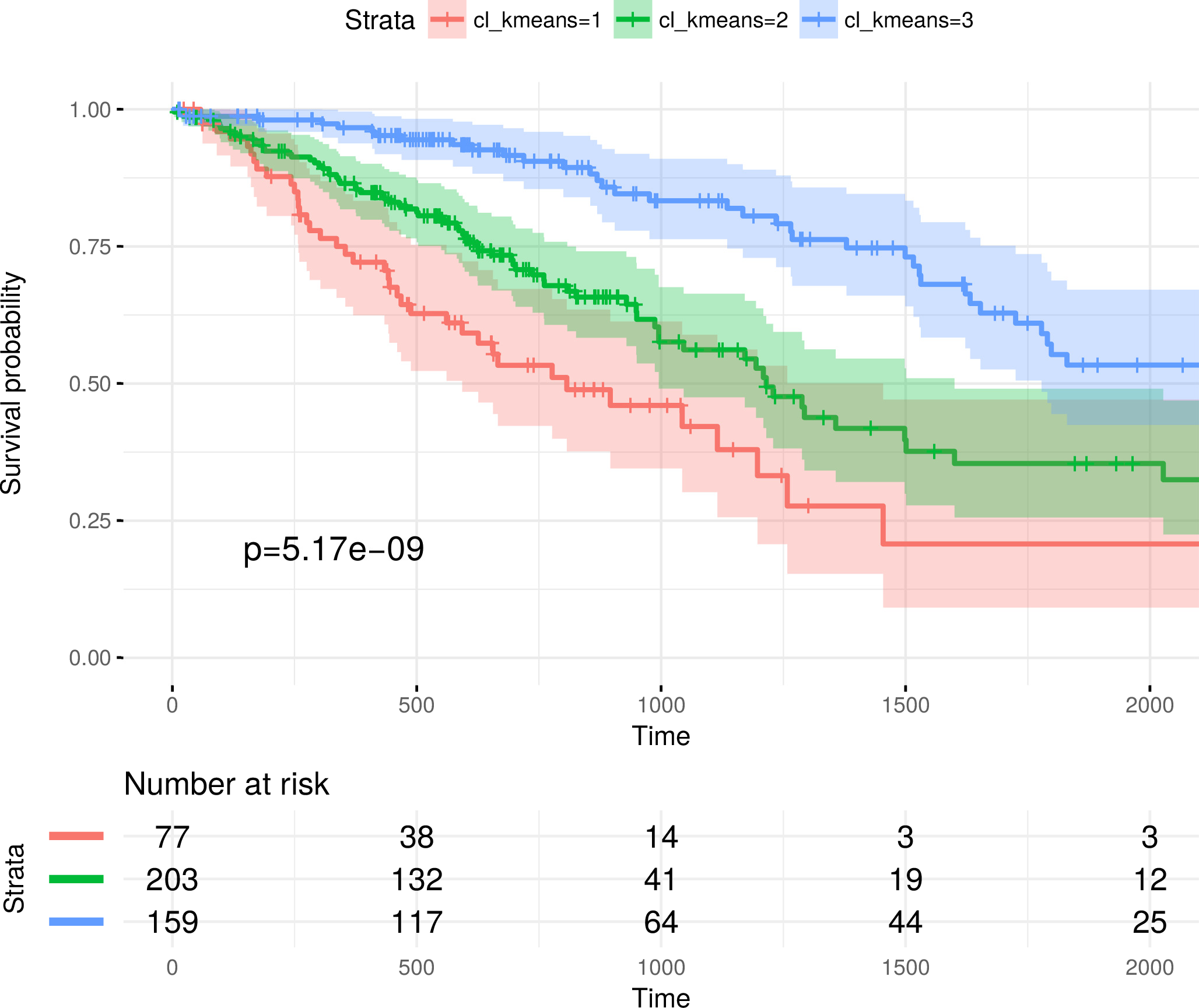

Supplement: S7 Fig — (TIF) [file pone.0204186.s007.tif]

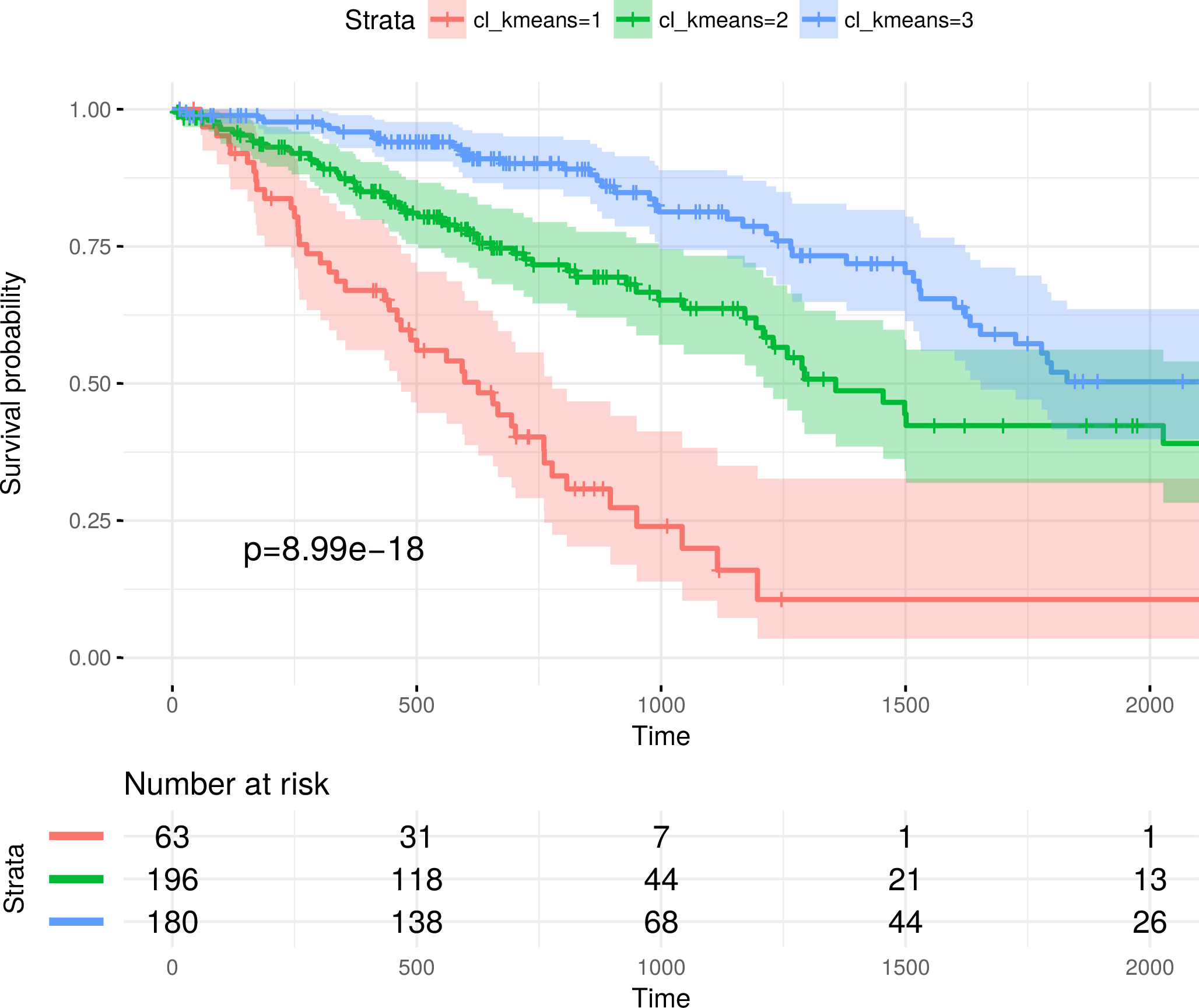

Supplement: S8 Fig — (TIF) [file pone.0204186.s008.tif]
