# Supplementary material for: EMT network-based feature selection improves prognosis prediction in lung adenocarcinoma
Source: PLoS One. 2019 Jan 31;14(1):e0204186. doi: 10.1371/journal.pone.0204186 (PMC6354965; doi:10.1371/journal.pone.0204186)
Supplement: S1 Table — All the following rules have confidence scores of 1. (PDF) [file pone.0204186.s009.pdf]

**Table 1. Top 20 prognostic association rules derived from the FSFs using filtered EMT network.** All the following rules have confidence scores of 1.

| | LHS | prognosis | supp | lift |
|---|---|---|---|---|
| 1 | $CDH3_{GE} = low, miR.34a_{DM} = high, HMGA2_{DM} = low$ | good | 0.113 | 1.956 |
| 2 | $EGLN3_{GE} = low, CDH3_{GE} = low, HMGA2_{DM} = low$ | good | 0.113 | 1.956 |
| 3 | $GFI1B_{GE} = high, CDH3_{GE} = low, HMGA2_{DM} = low$ | good | 0.135 | 1.956 |
| 4 | $GATA6_{GE} = low, E2F1_{GE} = low, HMGA2_{DM} = low$ | good | 0.120 | 1.956 |
| 5 | $GATA6_{GE} = high, CDC20_{GE} = low, FOXA3_{GE} = high$ | poor | 0.150 | 2.046 |
| 6 | $GATA6_{GE} = high, CDH3_{GE} = high, FOXA3_{GE} = high$ | poor | 0.135 | 2.046 |
| 7 | $GATA6_{GE} = high, miR.34a_{DM} = low, FOXA3_{GE} = high$ | poor | 0.143 | 2.046 |
| 8 | $miR.34a_{GE} = low, CCND1_{GE} = high, FOXA_{GE}3 = low$ | good | 0.113 | 1.956 |
| 9 | $GATA6_{GE} = low, miR.34a_{GE} = low, CCND1_{GE} = high$ | good | 0.105 | 1.956 |
| 10 | $BIRC3_{GE} = low, CCND1_{GE} = high, FOXA3_{GE} = low$ | good | 0.113 | 1.956 |
| 11 | $LOXL2_{GE} = high, miR.34a_{DM} = low, FOXA3_{GE} = high$ | poor | 0.158 | 2.046 |
| 12 | $BIRC3_{GE} = low, miR.34a_{GE} = low, CDH3_{GE} = low$ $HMGA2_{DM} = low$ | good | 0.105 | 1.956 |
| 13 | $GFI1B_{GE} = high, miR.34a_{GE} = low, HMGA2_{DM} = low$ $FOXA3_{GE} = low$ | good | 0.105 | 1.956 |
| 14 | $GFI1B_{GE} = high, BIRC3_{GE} = low, miR.34a_{GE} = low$ $HMGA2_{DM} = low$ | good | 0.105 | 1.956 |
| 15 | $ITGA6_{GE} = high, BIRC3_{GE} = high, BIRC5_{GE} = high$ $GATA6_{GE} = high$ | poor | 0.113 | 2.046 |
| 16 | $BIRC3_{GE} = high, GATA6_{GE} = high, E2F1_{GE} = low$ $miR.34a_{DM} = low$ | poor | 0.105 | 2.046 |
| 17 | $BIRC3_{GE} = high, GATA6_{GE} = high, E2F1_{GE} = low$ $GATA4_{GE} = low$ | poor | 0.135 | 2.046 |
| 18 | $BIRC5_{GE} = high, GATA6_{GE} = high, miR.34a_{GE} = high$ $FOXA3_{GE} = high$ | poor | 0.128 | 2.046 |
| 19 | $EGLN3_{GE} = high, BIRC5_{GE} = high, GATA6_{GE} = high$ $FOXA3_{GE} = high$ | poor | 0.113 | 2.046 |
| 20 | $BIRC5_{GE} = high, GATA6_{GE} = high, miR.192_{GE} = low$ $FOXA3_{GE} = high$ | poor | 0.120 | 2.046 |
