# Supplementary material for: EMT network-based feature selection improves prognosis prediction in lung adenocarcinoma
Source: PLoS One. 2019 Jan 31;14(1):e0204186. doi: 10.1371/journal.pone.0204186 (PMC6354965; doi:10.1371/journal.pone.0204186)
Supplement: S2 Table — We highlighted all p-values that are lower than 10e-5. (PDF) [file pone.0204186.s010.pdf]

Table 2. The p-values of log-rank tests based on the clustering of spectral clustering algorithm for different data level combinations using extended EMT network. We highlighted all p-values that are lower than 10e-5.

	GE	DM	CNA	GE+DM	GE+CNA	DM+CNA	GE+DM +CNA
t-test	1.19e-5	1.18e-1	6.39e-2	8.16e-1	**9.51e-7**	3.85e-1	1.86e-5
Lasso	2.34e-4	6.78e-1	7.67e-1	4.92e-1	1.76e-5	**1.85e-6**	**2.69e-6**
NetLasso	3.36e-2	8.61e-1	5.25e-1	2.96e-1	2.46e-2	2.61e-1	8.03e-1
addDA2	2.16e-5	4.55e-1	3.00e-4	**8.44e-13**	**3.09e-6**	**1.61e-7**	1.45e-5
NetRank	4.89e-03	1.82e-01	8.85e-01	5.30e-02	2.11e-02	1.62e-02	1.15e-01
stSVM	1.64e-01	5.22e-01	5.77e-01	5.62e-01	2.20e-01	7.45e-01	7.92e-01
Cox	3.49e-03	1.36e-04	1.04e-02	4.45e-05	1.34e-04	1.49e-05	2.10e-01
RegCox	1.41e-04	6.53e-03	1.60e-03	1.20e-01	7.63e-02	2.89e-01	4.07e-01
MSS	1.72e-03	8.71e-01	1.12e-01	4.51e-03	6.32e-04	1.59e-01	1.44e-02
Survnet	3.89e-05	2.48e-02	2.30e-01	9.20e-04	6.13e-04	1.23e-03	2.14e-05
Ensemble	1.40e-03	9.75e-01	8.37e-02	3.95e-03	**8.12e-07**	**2.42e-09**	**1.68e-08**
allemt	5.78e-03	8.37e-01	3.46e-01	9.58e-01	9.36e-03	7.79e-01	4.02e-01
