# Supplementary material for: EMT network-based feature selection improves prognosis prediction in lung adenocarcinoma
Source: PLoS One. 2019 Jan 31;14(1):e0204186. doi: 10.1371/journal.pone.0204186 (PMC6354965; doi:10.1371/journal.pone.0204186)
Supplement: S3 Table — We highlighted all p-values that are lower than 10e-5. (PDF) [file pone.0204186.s011.pdf]

Table 3. The p-values of log-rank tests based on SNF clustering using different data level combinations with extended EMT network. We highlighted all p-values that are lower than 10e-5.

	GE	DM	CNA	GE+DM	GE+CNA	DM+CNA	GE+DM +CNA
t-test	2.71e-05	6.33e-02	3.32e-01	6.81e-03	**5.61e-06**	2.22e-03	1.72e-03
Lasso	7.95e-04	9.79e-02	5.05e-01	4.39e-03	**8.30e-08**	1.87e-03	8.73e-03
NetLasso	7.09e-02	5.91e-01	2.51e-01	2.70e-01	2.38e-02	9.23e-02	7.39e-02
addDA2	**7.19e-07**	**8.27e-10**	8.50e-04	**9.60e-12**	1.36e-03	7.41e-02	**5.25e-09**
NetRank	2.05e-03	3.45e-01	3.17e-01	**1.61e-06**	1.09e-03	1.76e-03	2.07e-05
stSVM	9.83e-02	4.11e-01	5.76e-01	5.49e-01	5.58e-01	7.25e-01	6.61e-01
Cox	7.50e-05	9.18e-04	1.50e-01	3.33e-03	3.53e-03	4.47e-03	8.89e-04
RegCox	2.01e-03	6.60e-01	1.76e-01	**2.39e-08**	4.34e-02	**6.70e-06**	**1.28e-08**
MSS	9.45e-04	6.22e-02	3.47e-01	1.04e-02	1.63e-02	2.48e-01	6.00e-04
Survnet	9.44e-05	5.75e-02	3.14e-02	2.71e-03	4.95e-05	6.96e-05	9.74e-05
Ensemble	1.14e-03	4.10e-03	6.14e-02	1.20e-03	1.05e-02	5.73e-04	2.51e-04
allemt	1.94e-02	7.26e-01	2.70e-01	1.91e-01	3.41e-01	7.15e-01	5.92e-01
