# Supplementary material for: EMT network-based feature selection improves prognosis prediction in lung adenocarcinoma
Source: PLoS One. 2019 Jan 31;14(1):e0204186. doi: 10.1371/journal.pone.0204186 (PMC6354965; doi:10.1371/journal.pone.0204186)
Supplement: S4 Table — We highlighted all p-values that are lower than 10e-5. (PDF) [file pone.0204186.s012.pdf]

Table 4. The p-values of log-rank tests based on iCluster clustering using different data level combinations with extended EMT network. We highlighted all p-values that are lower than 10e-5.

	GE	DM	CNA	GE+DM	GE+CNA	DM+CNA	GE+DM +CNA
t-test	1.32e-02	3.16e-01	7.70e-02	1.24e-01	4.92e-04	6.65e-04	2.79e-01
Lasso	2.16e-01	8.66e-01	7.33e-01	1.26e-03	4.74e-05	1.64e-01	2.00e-04
NetLasso	4.23e-01	8.34e-01	7.63e-01	6.22e-01	3.75e-02	3.49e-01	4.62e-03
addDA2	1.69e-02	1.57e-02	4.23e-03	2.36e-01	3.48e-03	2.75e-02	2.74e-01
NetRank	2.41e-02	2.66e-01	4.33e-02	2.47e-02	5.04e-03	7.67e-02	7.71e-04
stSVM	**2.77e-08**	7.28e-01	1.33e-01	2.12e-01	7.46e-02	6.51e-01	9.37e-01
Cox	9.48e-04	6.85e-04	6.13e-01	4.01e-03	5.17e-04	3.07e-04	2.57e-02
RegCox	9.51e-01	2.61e-01	2.72e-02	2.22e-03	**6.12e-06**	3.57e-03	**2.96e-08**
MSS	3.76e-02	2.30e-01	1.33e-01	4.24e-03	5.75e-01	9.48e-01	2.06e-01
Survnet	1.25e-03	2.60e-01	6.94e-02	7.15e-01	2.71e-02	5.93e-02	4.51e-02
Ensemble	9.75e-03	8.77e-01	7.73e-02	2.50e-05	2.10e-03	1.10e-03	1.09e-03
allemt	7.15e-01	9.65e-01	2.68e-01	1.29e-01	7.58e-02	5.54e-01	1.70e-01
